# Use of gluteus maximus adipomuscular sliding flaps in the reconstruction of sacral defects after tumor resection

**DOI:** 10.1186/1477-7819-11-110

**Published:** 2013-05-23

**Authors:** Yao Weitao, Cai Qiqing, Gao Songtao, Wang Jiaqiang

**Affiliations:** 1 Bone and soft tumor department, He Nan Cancer Hospital, The Affiliated Hospital of Zheng Zhou University, 127 Dong Ming Road, Zheng Zhou City, 450000, China

**Keywords:** Sacral tumors, Surgery, Gluteus maximus adipomuscular sliding flaps

## Abstract

**Background:**

While performing sacrectomy from a posterior approach enables the en bloc resection of sacral tumors, it can result in deep posterior peritoneal defects and postoperative complications. We investigated whether defect reconstruction with gluteus maximus (GLM) adipomuscular sliding flaps would improve patient outcomes.

**Methods:**

Between February 2007 and February 2012, 48 sacrectomies were performed at He Nan Cancer Hospital, Zhengzhou City, China. We retrospectively examined the medical records of each patient to obtain the following information: demographic characteristics, tumor location and pathology, oncological resection, postoperative drainage and complications. Based on the date of the operation, patients were assigned to two groups on the basis of closure type: simple midline closure (group 1) or GLM adipomuscular sliding reconstruction (group 2).

**Results:**

We assessed 21 patients in group 1 and 27 in group 2. They did not differ with regards to gender, age, tumor location, pathology or size, or fixation methods. The mean time to last drainage was significantly longer in group 1 compared to group 2 (28.41 days (range 17–43 days) vs. 16.82 days (range 13–21 days, *P* < 0.05)) and the mean amount of fluid drained was higher (2,370 mL (range 2,000–4,000 mL) vs. 1,733 mL (range 1,500–2,800 mL)). The overall wound infection rate (eight (38.10%) vs. four (14.81%), *P* < 0.05) and dehiscence rate (four (19.05%)] vs. three (11.11%), *P* < 0.05) were significantly higher in group 1 than in group 2. The rate of wound margin necrosis was lower in group 1 than in group 2 (two (9.82%) vs. three (11.11%), *P* < 0.05).

**Conclusions:**

The use of GLM adipomuscular sliding flaps for reconstruction after posterior sacrectomy can significantly reduce the risk of infection and improve outcomes.

## Background

Sacral tumors include benign subtypes, such as giant-cell tumors [1] and aneurysmal bone cysts [2], as well as malignant subtypes, including chordomas, multiple myelomas and metastatic tumors [3]. These tumors are often asymptomatic or involve vague signs and symptoms [4], such as backache with or without numbness, leg weakness or bowel and/or bladder dysfunction. Therefore, diagnosis is frequently delayed by several months to up to 6 years [5]. During the interim, the tumors can become very large and may destroy most segments of the sacrum.

Treatment ranges from intralesional curettage [6] to ablation to *en bloc* excision [7]. Surgical resection can result in large defects that extend to the rectum ventrally or to the sacroiliac joints laterally. Defects can disrupt the posterior pelvic wall and often present a reconstructive challenge to surgeons [8]. The musculature over the sacrum can become weakened or of insufficient volume to fill large defects. Simple midline closures usually fail, which can lead to infection [9], wound breakdown, parasacral herniation or a combination of these complications, which may delay adjuvant treatment [10].

Good results have been reported with the use of vertical rectus abdominis myocutaneous flaps [11]. Other methods have involved meshes [12], omental mobilization [13] and free flaps [14]; however, some of these methods have been associated with additional injuries to patients and/or dangerous complications, such as necrosis [15]. We performed a retrospective study to assess whether reconstruction with gluteus maximus (GLM) adipomuscular sliding flaps would improve outcomes after sacral tumor excision.

## Methods

We assessed patients who had undergone sacrectomy because of sacral tumors between February 2007 and February 2012. All patients, or guardians when appropriate, gave informed consent before surgery. The authors obtained approval from the ethics committee of our hospital before doing the study.

**Figure 1:**
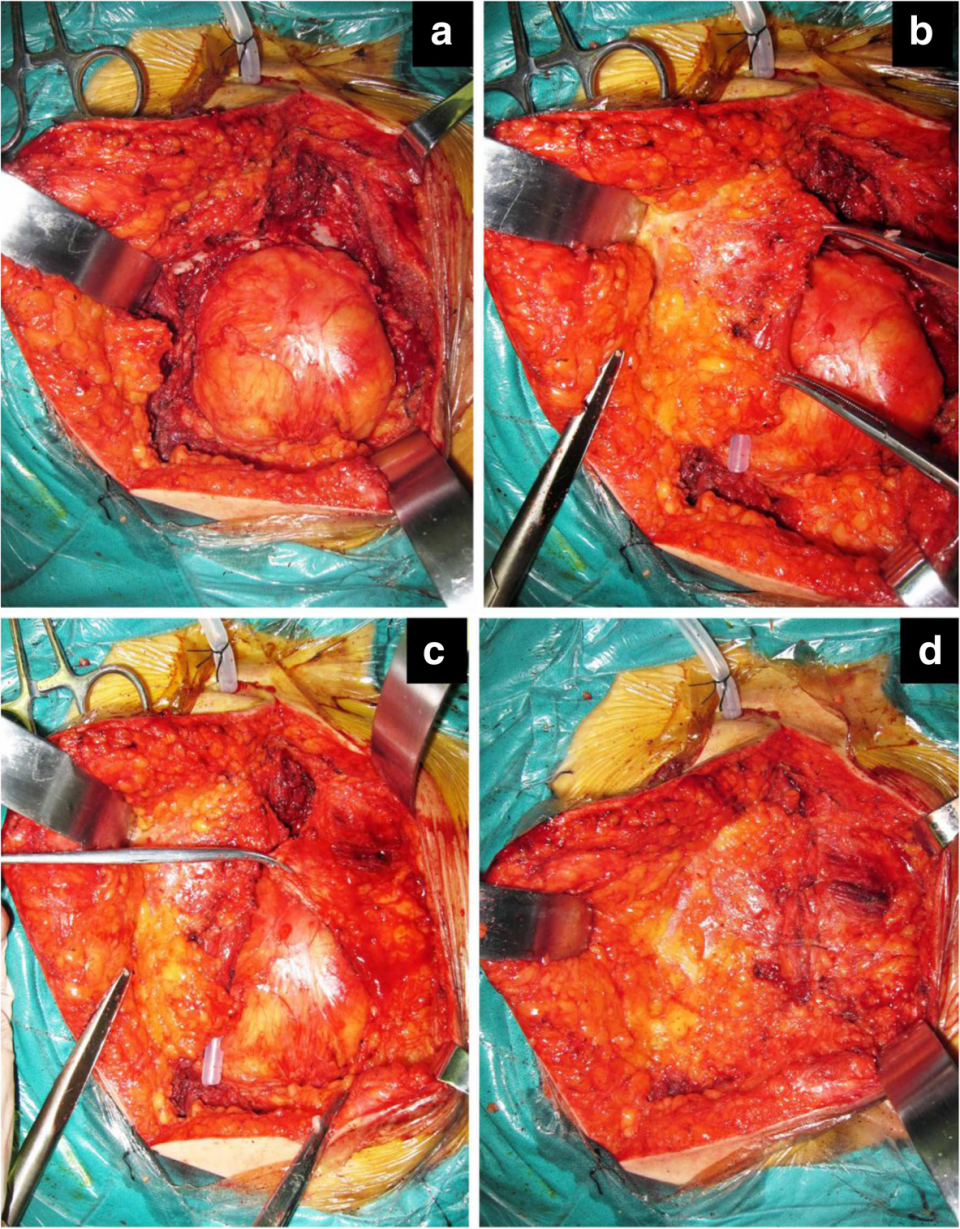
**Reconstruction of the sacral defect caused by tumor resection with bilateral gluteus maximus (GLM) adipomuscular sliding flaps.** A huge defect is left after sacral tumor resection (**a**). Separation of the GLM adipomuscular flap on one side (**b**). Separation of another side of the GLM adipomuscular flap (**c**). The defect is eliminated by advancement of the bilateral GLM adipomuscular flaps (**d**).

During sacrectomy, complete spondylectomies to the appropriate levels and sectioning of the bilateral piriform muscles and of the bilateral sacrospinous and sacrotuberous ligaments were carried out. The sacral nerve roots (above sacral vertebra S3) of involved segments on both sides were separated and protected (Figure 1a). If total sacrectomy was performed, complex lumbopelvic reconstruction and arthrodesis were undertaken with autologous iliac crest bone grafts and instrumentation (for example, bilateral lumbar vertebrae L4 and L5 pedicle screws and iliac screws). Blood loss was controlled by temporary abdominal aorta block during sacrectomy of S1.

**Figure 2:**
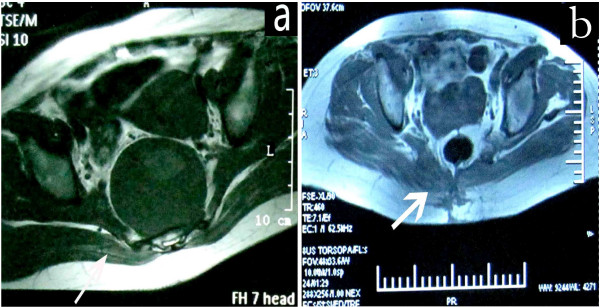
**Magnetic resonance imaging of the tumor and flap.** Sacral tumor and intact gluteus maximus (GLM) (arrow) before surgery (**a**). Well-maintained GLM adipomuscular flap (arrow) behind the rectum (**b**) 6 months after surgery.

Based on the date of the operation, from February 2007 to December 2010, patients who underwent simple midline closure by two-layer suture of the fascia and skin comprised group 1. From January 2011 to January 2013, patients were chosen for GLM adipomuscular sliding flaps reconstruction if they had undergone resection of a sacral tumor via an exclusively posterior approach, had no tumor invasion on either side of the GLM muscle on MRI before surgery (Figure 2a), had adequate blood supply to the GLM flap and had not undergone previous sacral radiotherapy. These patients comprised group 2.

One or both sides of the GLM adipomuscular sliding flaps were used to fill the dead space at the posterior peritoneum defect depending on the invasion and resection of GLM (see Figure 2a). In cases where both sides of the GLM were intact with no resection during the operation, bilateral flaps were used to fill the defect. When the GLM was invaded by tumors and wide resection was undertaken, the other side of the muscle flap was used to slide into the defect.

The first step was elevating the gluteal skin flap, including the superficial fascia, based on the lumbar vascular perforation. The deep adipose tissue beneath the superficial fascia was left on the GLM muscle (see Figure 1b and c). The separation area included the following: the upper boundary of the flap reaching the iliac crest, the low boundary of the buttock line according to the area of the posterior peritoneal defect, and the 6 to 10 cm of adipomuscle that lay in between. Then, the unilateral or bilateral adipomuscular flaps were slid into the retroperitoneal space and sutured together in the midline to obliterate the dead space (see Figure 1d). Wound drainages were placed into the bone defect and the subcutaneous cave of all patients after surgery.

All patients ate a normal diet after surgery. Early ambulation was allowed in patients whose procedures did not involve instrumentation. By 2 weeks post-surgery, patients without instrumentation were allowed to get out of bed supervised by a physiotherapist. If patients had undergone lumbopelvic reconstruction and arthrodesis, weight-bearing activity was delayed until new bone formation was seen at the sites of bone grafting. Gait was evaluated with the following grades: normal, slight limp and severe limp. Movement of hip joints was measured and recorded.

Wound drainage was continued in all patients for at least 2 weeks. Drains were removed when the output was 5 mL or less for two consecutive days. The number of days to final drainage and the amount of fluid drained were both recorded. Chemotherapy or radiotherapy were used to treat multiple myelomas or metastatic tumors after wound healing.

Postoperatively, all patients were discharged from the hospital when the wound healed and the drains were removed. They were followed up on a monthly basis for 3 months, and were then seen every 3 months for 2 years, and every 6 months for years 3 to 4. At each visit, patients underwent radiography of the sacral area and physical examination. Magnetic resonance imaging (MRI) was used to assess local tumor collapse 12 to 48 months post-operation in selected patients who had giant-cell tumors, chordomas, multiple myelomas and metastatic tumors.

We recorded the sex, age, sacral segment, pathology, size of tumor and resection and fixation methods in a coded spreadsheet. Statistical analysis was conducted with the SPSS statistical software package (version 11.5; Chicago, IL, USA). Sex, age, sacral segment, pathology, size of the defect, the type of resection methods and reconstruction, postoperative complications (infection, flap necrosis, wound dehiscence and parasacral hernia) and functional outcomes (such as gait) were compared between groups 1 and 2 with a nonparametric independent-samples *t*-test. The movement of hip joints, mean time to last drainage and the mean total amount of fluid drained were compared with an independent-samples *t*-test. A difference with *P* <0.05 was considered to be statistically significant.

## Results

The patient population consisted of 32 men and 16 women, with a mean age of 56.82 years and a range of 23 to 83 years. The histological diagnoses and sacral segments removed are shown in Table 1. Group 1 comprised 21 patients and group 2 comprised 27 patients. The groups did not differ significantly with regards to demographic or pathological characteristics, size of tumor or fixation (Table 1). The mean diameter of sacral defects was 17.4 cm (range 6.5 to 35.8 cm). In group 2, bilateral GLM adipomuscular sliding flaps were used in 23 patients, while unilateral GLM adipomuscular sliding flaps were used in 4 patients. The flaps enabled the successful reconstruction of the posterior peritoneal defect, and the volume of the flap was well maintained behind the rectum on postoperative MRI in all cases (Figure 2b).

**Table 1:** Characteristics of patients grouped by closure method

	**Group 1**	**Group 2**	***P-*value**
Gender			
Male	13 (61.90%)	19 (70.37%)	0.85
Female	8 (38.10%)	8 (29.63%)	
Age, years			
20 to 40	3 (14.29%)	5 (18.52%)	0.06
40 to 60	11 (52.38%)	16 (59.26%)	
60 to 80	7 (33.33%)	6 (22.22%)	
Segment of sacrum			
S4 to S5	1 (4.76%)	1 (3.70%)	0.06
S4 to S3	2 (9.82%)	4 (14.81%)	
S2 to S3	6 (28.57%)	7 (25.93%)	
S1 to S2	7 (33.33%)	10 (37.04%)	
L5 to S1	5 (23.81%)	5 (18.52%)	
Pathology			
Chordomas	12 (57.14%)	14 (51.85%)	0.05
Multiple myelomas	2 (9.52%)	3 (11.11%)	
Metastatic tumors	2 (9.52%)	5 (18.52%)	
Giant-cell tumor	4 (19.05%)	3 (11.11%)	
Others	1 (4.67%)	1 (3.70%)	
Size of tumor, diameter			
<10 cm	11 (52.38%)	6 (22.22%)	0.11
10 to 20 cm	7 (33.33%)	14 (51.85%)	
>20 cm	3 (14.29%)	7 (25.93%)	
Resection methods			
Radical resection	10 (47.62%)	14 (51.85%)	0.02
Marginal resection	6 (28.57%)	9 (33.33%)	
Intradural curettage	5 (23.81%)	4 (14.81%)	
Fixation			
Yes	10 (47.62%)	14 (51.85%)	0.84
No	11 (52.38%)	13 (48.15%)	

Data are number (%).

Four patients died as a result of the primary disease within 9 to 24 months, and seven patients had a relapse at the site of the operation during the 10 to 32 month follow up period in the two groups. The mean time to final drainage and the mean amount of fluid drained differed significantly between the two groups, with better outcomes seen in group 2 (Table 2). Eight patients in group 1 and four patients in group 2 developed wound infections 2 to 4 weeks after surgery, and this difference was statistically significant (Table 2). The rate of wound dehiscence was lower in group 2 than in group 1 (three (11.11%) versus four (19.05%), *P* <0.05). However, margin necrosis was more frequent in group 2 than in group 1 (three (11.11%) versus two (9.82%), *P* <0.05). Gait and hip joint movement were preserved in all cases, irrespective of closure method.

**Table 2:** Surgical outcomes according to treatment group

**Drainage and complications**	**Group 1**	**Group 2**	***P-*value**
Time to last drainage, days	28.41 ± 11.05	16.82 ± 7.38	0.02
Amount of fluid drained, mL	2370 ± 284	1733 ± 326	0.00
Wound infection	8 (38.10%)	4 (14.81%)	0.00
Wound margin necrosis	2 (9.82%)	3 (11.11%)	0.00
Wound dehiscence	4 (19.05%)	3 (11.11%)	0.00

Data are mean ± SD or number (%).

## Discussion

As sacral tumors are often asymptomatic or the symptoms are vague (low-back pain with or without numbness, leg weakness or bowel and/or bladder dysfunction), diagnosis is frequently delayed by several months to several years, depending on the rate of tumor growth [5]. Thus, at the time of diagnosis, the sacral roots might be involved or even have been destroyed. Tumors can become very large and extend into the surrounding soft tissue, including the GLM muscles, the presacral space and beyond the sacroiliac joints to the ileum.

The first-line treatment for sacral tumors is surgery, and is often radical, or may involve marginal surgical excision. Extensive resection, however, always leaves a large defect, usually with a diameter of 6.5 to 35.8 cm that extends laterally from the sagittal lumbar spine to the sacroiliac joints. In these cases, simple midline closures generally fail and have a high risk of wound infection or dehiscence. Development of successful techniques for reconstruction of these defects is, therefore, important to lower the risk of complications.

Many techniques have been used for reconstruction after sacrectomy, including myocutaneous flaps, free flaps and mesh. The most commonly used is trans-pelvic vertical rectus abdominis myocutaneous flaps, which involves a circumferential approach. This method has several advantages, including the ease of the procedure, providing suitable bulk, a long pedicle, adequate blood supply and re-creation of the pelvic floor [16]. Use of this method in conjunction with a laparotomy or ostomy, however, potentially weakens the anterior abdominal wall and causes incisional hernia [17]. The use of free flaps has been described for lumbosacral reconstruction when local flaps are destroyed. The first-choice recipient vessels are the superior or inferior gluteal vessels because they lay in or beside the defect [18]. If these vessels are unavailable, the femoral and thoracodorsal vessels may be used; however, this approach requires long vein grafts and potentially increases the risk of skin-flap necrosis [19]. Koh PK *et al*. [20] have reported the use of the GLM muscle turnover flaps for reconstruction of sacral chordoma defects. The advantages of using the GLM muscle are its bulk, proximity to the defect and robust blood supply. The disadvantages of this approach include the necessity of an additional incision, destruction of some of the GLM blood supply and the risk of gluteal gait.

The blood supply of the GLM muscle consists of the superior and inferior gluteal arteries, which communicate with perforating branches from the femoral system and the medial femoral circumflex arteries to form an intricate cruciate anastomosis around the hip. Because of this rich blood supply, the use of GLM flaps is feasible for defect reconstruction in selected patients. With the adipomuscular sliding flaps technique, the muscles are not elevated but are simply advanced toward the midline, which leaves their insertions untouched. The suturing of each muscle to its contralateral counterpart creates a strong posterior peritoneal repair that employs the blood supply of both the superior and inferior pedicles. During the operation, none of the blood vessels need to be exposed or elevated, which makes this flap operation safe and easy. This method, however, yields less flap volume and less moving distance than the other GLM flap approaches reported previously [20], and is only suitable for defects less than 30 cm in diameter. This type of flap surgery was performed in all group 2 patients in this study because of its convenience and because most sacral tumors have less invasion to the GLM muscle.

The use of GLM adipomuscular sliding flaps has many advantages over other reconstruction and closure approaches. As well as the robust blood supply mentioned above, the GLM muscle is large and the anatomic location is proximal to the sacrum. Moreover, no additional intraoperative incisions or repositioning of the patient are required. When a sacrectomy is performed from a posterior approach, the three main blood supply routes are not routinely sacrificed. The retention of a native and robust blood supply may be central to the long-term durability and viability of flaps. The separation and movement of flaps is much easier than other kinds of GLM flap approaches, such as the use of antegrade or retrograde GLM rotation flaps described by Koh *et al*. and Vogt *et al*. [19,20]. The bulk achieved with GLM adipomuscular sliding flaps helps protect the blood supply and makes these approaches ideal for sacral defects.

Our results show that in group 2, both the time to final drainage and the amount of fluid drained were significantly reduced compared to group 1. The rates of infection and wound dehiscence were also significantly lower in group 2 relative to group 1. GLM adipomuscular sliding flaps appear, therefore, to form a protective layer while also absorbing cavity effusion, which may be the reasons underlying the reduced rate of perianal infection. Because of the poor blood circulation in subcutaneous tissue of the gluteal groove, however, flap necrosis cannot be improved.

Since the GLM muscle is the primary extensor of the hip, the potential drawbacks of using GLM flaps are functional disturbance in ambulation, especially when walking upstairs or straightening from a bending position, and pelvic instability [21]. We found no difference between the two groups with regards to ambulatory status. This finding might have arisen because the bilateral advancement flap technique was used in most patients, which does not hinder active extension of the hip and is compensated by gradual hypertrophy of the hamstring muscle and the adductor magnus. Other potential adverse side effects that come with use of GLM adipomuscular sliding flaps include poor extensibility, which can lead to excessive traction on the flap. Additionally, the flaps may not have enough bulk to cover the defect, especially if most of the GLM muscle on one side needs to be removed.

## Conclusions

Our experience suggests that reconstruction with GLM adipomuscular sliding flaps improves outcomes after sacrectomy. The use of unilateral or bilateral GLM adipomuscular sliding flaps should therefore be considered in patients who have not undergone prior radiation therapy, who have intact GLM muscles with gluteal vessels on one or both sides and who have defects less than 30 cm in diameter. This technique had low rates of complications and morbidity, was easy to perform, and had high success rates.

## Competing interest

The authors declare that they have no competing interests.

## Authors’ contribution

All authors read and approved the final manuscript.
